# Feasibility and Acceptability of an Adapted Mobile Phone Message Program and Changes in Maternal and Newborn Health Knowledge in Four Provinces of Afghanistan: Single-Group Pre-Post Assessment Study

**DOI:** 10.2196/17535

**Published:** 2020-07-20

**Authors:** Victoria Lebrun, Lisa Dulli, Sayed Omar Alami, Arzoo Sidiqi, Ahmad Shah Sultani, Sayed Haroon Rastagar, Iftikhar Halimzai, Sharif Ahmadzai, Catherine S Todd

**Affiliations:** 1 Global Health, Population, and Nutrition FHI 360 Durham, NC United States; 2 Helping Mothers and Children Thrive in Afghanistan (HEMAYAT) Project FHI 360 Kabul Afghanistan; 3 Health Promotions Department Ministry of Public Health Kabul Afghanistan

**Keywords:** Afghanistan, mobile apps, pregnant women, maternal health, newborn health, social and behavior change, mHealth, voice message, SMS

## Abstract

**Background:**

Mobile phone apps for health promotion have expanded in many low- and middle-income countries. Afghanistan, with high maternal and newborn morbidity and mortality rates, a fragile health infrastructure, and high levels of mobile phone ownership, is an ideal setting to examine the utility of such programs. We adapted messages of the Mobile Alliance for Maternal Action (MAMA) program, which was designed to promote healthy behaviors during pregnancy and a newborn’s first year of life, to the Afghan context. We then piloted and assessed the program in the provinces of Kabul, Herat, Kandahar, and Balkh.

**Objective:**

The aim of this study was to assess the feasibility and acceptability of the MAMA pilot program, and to examine changes in reported maternal, newborn, and child health (MNCH) knowledge and attitudes among participants from baseline to follow up.

**Methods:**

We conducted a single-group study with data collected within 10 weeks of enrollment, and data collection was repeated approximately 6 months later. Data were collected through face-to-face interviews using structured questionnaires. Eligible participants included pregnant women who had registered to receive fully automated mobile health messages and their husbands. Assessment questionnaires queried sociodemographic details; knowledge, attitudes, and health care-seeking practices; and intervention experience and acceptability at follow up. The number of messages received by a given phone number was extracted from the program database. We descriptively analyzed the feasibility and acceptability data and compared the change in MNCH knowledge between baseline and follow-up measures using the McNemar Chi square test.

**Results:**

Overall, 895 women were enrolled in the MAMA program. Data from 453/625 women (72.5% of the pretest sample) who received voice (n=302) or text (n=151) messages, and 276/427 men (64.6% of the pretest sample) who received voice (n=185) or text (n=91) messages contributing data at both time points were analyzed. At follow up, 699/729 (95.9%) participants were still enrolled in the MAMA program; voice message and SMS text messaging subscribers received 43 and 69 messages on average over the 6-month period, respectively. Participants who were voice message subscribers and female participants more commonly reported missing messages compared with the text message subscribers and men; predominant reasons for missed messages were the subscribers being busy with chores or not having their shared phone with them. Over 90% of men and women reported experiencing benefits from the program, mainly increased knowledge, and 226/453 (49.9%) of the female participants reported referring someone else to the program. Most of the participants (377/453, 83.2% women and 258/276, 93.5% men) believed it was beneficial to include husbands in the program. Joint decision making regarding maternal and child health care increased overall. The proportions of participants with correct knowledge significantly increased for all but one MNCH measure at follow up.

**Conclusions:**

This assessment indicates that the pilot MAMA program is feasible and acceptable in the Afghan context. Further research should be conducted to determine whether program participation leads to improved MNCH knowledge, health practices, and health service utilization in this fragile setting prior to larger scale up.

## Introduction

Afghanistan has one of the highest maternal, newborn, and child mortality rates among Asian countries [1] and faces important challenges to improving these and other health indicators [2]. Despite substantial investment in training and deploying large numbers of health workers, there is uneven geographic and socioeconomic coverage of essential interventions in the country, including antenatal care, skilled birth attendance, immunization, and family planning [3]. Uptake of these services, where available, is also suboptimal. Only 18% of women had at least 4 antenatal care visits and 51% reported skilled birth attendance at their last delivery in the 2015 Demographic and Health Survey [4]. A 2013 survey found that 51% of children aged 12-23 months were fully immunized based on Afghanistan’s national immunization schedule [5]. Additionally, the contraceptive prevalence rate has stagnated at approximately 20% since 2010 [3,4].

Among the factors associated with low uptake of essential maternal, newborn, and child health (MNCH) interventions are illiteracy and lack of education [6]. Maternal and infant morbidity and mortality rates are disproportionately higher among rural populations, which have lower literacy levels and are less likely to access health services or to be reached by health messaging through mass media or health providers [4,7,8]. Women’s access to health services is also hampered by traditional social norms, which position men as the primary health decision makers and limit women’s freedom of movement [9,10]. Health decision making occurs within the family structure with men, and sometimes older women such as mothers-in-law, serving as gatekeepers who determine when and where health services for pregnant women and infants are sought [5,11-13].

Within Afghanistan, mobile phone ownership and use have expanded markedly in the last decade, creating a potential channel to reach women and their families with health information. In a recent nationally representative survey, 91% of households reported owning a mobile phone [14]. Mobile phone ownership is higher among men compared to women; however, a 2012 cross-sectional survey found that 80% of women reported routinely using a mobile phone [15], and evidence suggests that their phone use is increasing. A 2015 cross-sectional survey in Nangarhar province found that 92% of women routinely used mobile phones [16]. Mobile phone–based programming for various health education purposes has been documented globally with mixed success; however, to date, there is no literature on the use of such programs in Afghanistan. Short message service (SMS) text messaging recommendations and appointment reminders during pregnancy have been positively received and were shown to improve antenatal and postnatal care attendance in other low- and middle- income countries [17-20]; recorded voice messages in addition to, or in place of, SMS text messaging enable low literacy populations to access health information [21]. The 2019 World Health Organization guideline “Recommendations for digital interventions for health system strengthening” recommends targeted client communications through digital channels, including mobile phone messages, to promote reproductive, maternal, and newborn health [22].

The Mobile Alliance for Maternal Action (MAMA) program was launched in 2012 as a public-private partnership to scale up an evidence-based mobile health (mHealth) program that is already being used in two countries [23]. With support from Baby Center, Johnson & Johnson, the United States Agency for International Development (USAID), the mHealth Alliance, and the United Nations Foundation [23-25], MAMA implemented its mHealth approach focused on age- and stage-based messaging directed toward pregnant women and their family members in Bangladesh [24,25], India, South Africa [26], and Nigeria. The MAMA program relays essential MNCH messages promoting healthy practices and care-seeking for pregnant women and their infants up to 12 months of age [23]. These messages are available for adaptation and use through an application process to interested groups in different countries. Countries employing the program have developed context-appropriate business models and message delivery channels, and have also identified and targeted subpopulations for program promotion [23].

In this study, we assessed a pilot implementation of the MAMA program in four provinces of Afghanistan to determine whether it was feasible and acceptable to Afghan users, and whether users report changes in MNCH attitudes, health decision making, and knowledge of key MNCH concepts. The data from this assessment will guide program refinement, and are intended to improve program use and potential effectiveness at scale up in Afghanistan and adaptation in similar contexts.

## Methods

### Study Design

We conducted a single-group, baseline/follow-up study to assess the feasibility, acceptability, and potential effect of the MAMA program on MNCH knowledge. The MAMA pilot program was implemented in 80 health post catchment areas in the Balkh, Herat, Kabul, Kandahar, and Nangarhar provinces of Afghanistan. Nangarhar province was not included in the program assessment as baseline recruitment was not possible within the study time frame due to insecurity. The selected pilot districts were semiurban or rural and predominantly agrarian. Eligible study participants included pregnant women and their husbands who enrolled in the MAMA program with the help of a community health worker between May and July 2018.

The study was reviewed and approved by the FHI 360 Protection of Human Subjects Committee (#1240522-2) and was approved by the Afghanistan Ministry of Public Health (MOPH) Institutional Review Board (protocol #444670) prior to implementation.

### Program Design

The MAMA message program relays essential MNCH information to guide actions for pregnant women and families with children under 12 months of age through mobile phones. Educational messages are sent twice weekly and are timed to the stage of pregnancy or age of the newborn. In a report exploring MAMA program implementation experiences in four countries, several factors were noted that may impact feasibility and acceptability [23]. Specifically, the report highlights the need to adapt the content to specific contexts with local stakeholder inputs; customer enrollment requires partners with local presence; and the need for client-centered message delivery, such as selecting the best time for calls [23]. In 2018, MAMA program messages were adapted to the Afghan context by technical specialists in social and behavior change, MNCH, and community health from the USAID-funded Helping Mothers and Children Thrive in Afghanistan (HEMAYAT) project, the Health Promotions and Reproductive Health Departments of the Afghanistan MOPH, and members of the Health Promotions Technical Working Group. The resulting adapted and translated messages were recorded by female native Dari and Pashto speakers as voice messages, pretested, and revised. Following pretesting, final messages were reviewed and approved by the Health Promotions Technical Working Group. An automated messaging platform was developed by Parsa Technology in Kabul, Afghanistan, which allowed user selection of preferred time of day to receive twice-weekly messages, message type (voice message or SMS text message), and language (Dari or Pashto) that then sent messages to subscriber phone numbers across a variety of mobile networks.

For pilot program implementation, female community health workers were selected from communities with active health posts. Each selected community health worker was trained to recruit and enroll 10 pregnant women and offer enrollment to their husbands, in collaboration with the Provincial Health Directorate and the Basic Package of Health Services implementer. Recruitment was primarily achieved through home visits to prospective clients, although some participants were recruited at locations such as health posts and adult literacy classes. For this pilot program, eligibility for registration was confined to pregnant women and their husbands who lived within the catchment area of the health post of the recruiting community health worker and had access to a functional mobile phone. Potential clients were informed that program staff might call them to confirm message receipt and program functionality. The community health workers ensured that women and men who registered were eligible and provided clients with a choice of written text (SMS) messages or automated recorded voice calls, as well as a choice for time of day to receive the calls or text messages. If the couple shared a phone, the woman was offered these choices, as she was considered the primary subscriber. The first message was timed to the woman’s current week of pregnancy and continued sequentially from that point. Messages encouraged clients to discuss MNCH care with their spouses and families, and promoted healthy practices such as exclusive breastfeeding and seeking health care services, including antenatal care, skilled attendance at delivery, and infant immunization.

Monitoring was conducted by HEMAYAT program staff and community health supervisors through three approaches: direct field supervision and mentoring during the community health worker-led registration process; monitoring of the data dashboard incorporated with the MAMA platform reflecting the number of messages sent, received, and nonresponsive numbers; and project staff calls to registered clients to confirm number validity, message receipt, and whether the registered client met the criteria for the pilot registration process. As the first and third approaches were employed early in pilot program implementation, system errors such as some mobile networks not receiving messages were identified and repaired.

### Sampling and Recruitment

For baseline data collection, all households that had enrolled in the MAMA program in the four included provinces were approached and program subscribers were recruited to participate in the study no more than 10 weeks after enrolling in the MAMA program. The community health workers who had promoted the program and registered subscribers introduced the data collection team to female subscribers and their husbands or male heads of household. Eligible participants were pregnant women and their husbands subscribing to the pilot program at least 4 weeks earlier and verbally consenting to study participation. Following explanation of the assessment, participation was offered, and verbal informed consent was obtained from subscribers interested in study participation by data collectors of the same sex. For follow-up data collection, households were revisited approximately 6 months after the baseline survey.

### Measures and Data Collection

Structured interviews using paper questionnaires were conducted with study participants by trained, sex-matched data collectors fluent in the language(s) predominantly spoken in that province. Translated questionnaires for women and men were field-tested with volunteers from different ethnic groups across implementation sites. The baseline questionnaire included sections detailing household characteristics, the program registration process, and MNCH-related knowledge and attitudes. Interviewers asked program participants about their attitudes toward discussing MNCH and specific practices, household decision making about MNCH care, and their knowledge of several maternal and newborn health issues featured in MAMA messages. We developed the knowledge questions based on content used to measure MNCH knowledge among men and women in assessments and evaluations performed in Afghanistan both within our group and in larger household-level surveys [4,27]. Knowledge questions were closed-ended and were in the form of single-response (for questions that have one correct answer such as the number of months for which infants should be exclusively breastfed) or multiple-response (for questions with more than one correct answer, such as serious health problems that can occur during pregnancy) questions. Answer choices were not read to the participant. Knowledge scores for multiple-response questions were converted to a binary score with 1 assigned to any correct answer and 0 assigned to no correct answers.

Feasibility measures included the proportion of female respondents who had to ask permission to register and from whom they asked permission; whether they were asked what time of day they prefer to receive messages and whether they received messages at their preferred time; continued to subscribe and receive messages after 6 months and the number of messages that respondents reported receiving per week on average; and reports of missed messages with stated reasons for missing the MAMA messages. Acceptability measures included the proportion of respondents who reported any and specific benefits to their participation in MAMA; reported that a member of their household listened to or read the messages and who it was (eg, husband, mother-in-law); stated that including their husband or their mother-in-law was beneficial; recommended MAMA use to someone else; and discussed MAMA with other pregnant women or new mothers.

Trained male and female data collectors administered the study instruments to consenting participants of the same sex in a private room in the household. The same data collectors conducted interviews at baseline and follow-up sessions; community health workers introduced the data collection team at households during study recruitment and at baseline data collection, consistent with cultural norms. Baseline data were collected in July and August 2018. Follow-up interviews were conducted with the same participants in January and February 2019, using a similar structured questionnaire including the same knowledge and attitude questions and adding questions on exposure to and acceptability of the MAMA program. Additionally, the MAMA mobile platform and database automatically recorded the dates and time of messages that were sent and received. We extracted the overall number of messages received for each participant at follow-up data collection.

### Data Analysis

We used STATA Version 15 (StataCorp LP, College Station, TX, USA) to descriptively analyze sociodemographic characteristics, message exposure, and acceptability measures, disaggregated by sex and message type (SMS text or voice message). Results were summarized at baseline and at follow up for attitudes, decision making, and knowledge of MNCH topics. Additionally, we conducted exploratory analyses to examine the change in selected MNCH knowledge measures between baseline and follow up using the McNemar Chi square test for paired data, with a two-sided alpha of .05 and 80% power.

### Data Availability

The datasets generated and analyzed during the current study are available from the corresponding author on reasonable request.

## Results

### Participant Characteristics

Across the four provinces sampled, 895 pregnant women were registered in the MAMA pilot program from May to August 2018. Over two-thirds (625/895, 69.8%) of female program participants and 427 husbands who were confirmed subscribers to the program agreed to participate in the study. A total of 499/625 (79.8%) women and 306/427 (71.7%) men completed both baseline and follow-up questionnaires. In some households, more than one woman or man enrolled in the study. However, because telephone numbers were used as the unique identifier to link baseline and follow-up data for participants, if more than one person of the same sex had the same phone number, it was impossible to link the data from the two time points to the correct participant. Thus, due to this uncertainty, we removed records for both participants from the final dataset. Therefore, we present results from 453/625 women (72.5% of the pretest sample) and 276/427 men (64.6% of the pretest sample) with complete baseline and follow-up data, disaggregated by sex.

Among both women and men, about two-thirds of participants (487/729, 66.8% ) opted to receive voice messages and one-third (242/729, 33.2%) chose to receive SMS text messages. Sociodemographic and household characteristics of participants indicate that many women did not own a phone and used someone else’s phone to access the program, and most of the participants had primary-level or no education (Table 1). Over three-quarters of women who chose voice messages reported having no formal education, compared to one-third of women who selected SMS text messages. High numbers of SMS text message recipients reported having electricity, televisions, and mobile phones with internet access in their households compared to participants who chose to receive voice messages.

**Table 1:** Sociodemographic characteristics of participants, by sex and message modality, across four provinces in Afghanistan (N=729).

Characteristic		Women		Men	
		Voice (n=302)	Text messaging (n=151)	Voice (n=185)	Text messaging (N=91)
**Province, n (%)**					
	Balkh	77 (25.5)	56 (37.1)	58 (31.4)	42 (46.2)
	Herat	73 (24.2)	48 (31.8)	36 (19.5)	29 (31.9)
	Kabul	66 (21.9)	38 (25.2)	28 (15.1)	12 (13.2)
	Kandahar	86 (28.5)	9 (6.0)	63 (34.1)	8 (8.8)
Age (years), mean (SD)		28.6 (12.7)	26.3 (10.9)	32.5 (9.6)	30.5 (7.5)
Phone ownership, n (%)		176 (58.3)	104 (68.9)	185 (100.0)	88 (96.7)
**Phone used to access MAMA^a^, n (%)**					
	Own	123 (40.7)	70 (46.4)	134 (72.4)	57 (62.6)
	Spouse’s	146 (48.3)	67 (44.4)	42 (22.7)	29 (31.9)
	Mother/father’s	1 (0.3)	6 (4.0)	3 (1.6)	3 (3.3)
	Mother-in-law/father-in-law’s	20 (6.6)	1 (0.7)	3 (1.6)	0 (0)
	Brother/sister’s	2 (0.7)	5 (3.3)	1 (0.5)	2 (2.2)
	Brother-in-law/sister-in-law’s	7 (2.3)	2 (1.3)	0 (0)	0 (0)
**Education level, n (%)**					
	No formal education	234 (77.5)	46 (30.5)	84 (45.4)	29 (31.9)
	Primary	31 (10.3)	25 (16.5)	38 (20.5)	12 (13.2)
	Secondary	25 (8.3)	25 (16.6)	19 (10.3)	14 (15.4)
	High school	10 (3.3)	40 (26.5)	25 (13.5)	21 (23.1)
	Higher	1 (0.3)	13 (8.6)	12 (6.5)	12 (13.2)
	Vocational or Madrassa	1 (0.3)	2 (1.3)	7 (3.8)	3 (3.3)
**Number of living children at pretest, n (%)**					
	0	53 (17.6)	37 (24.5)	39 (21.1)	26 (28.6)
	1-2	82 (27.2)	56 (37.1)	40 (21.6)	35 (38.5)
	3-4	84 (27.8)	40 (26.5)	54 (29.2)	19 (20.9)
	5 or more	83 (27.5)	18 (11.9)	52 (28.1)	11 (12.1)
**Household wealth status indicators^b^, n (%)**					
	Electricity	228 (75.5)	136 (90.1)	153 (82.7)	85 (93.4)
	Radio	72 (23.8)	36 (23.8)	69 (37.3)	24 (26.4)
	Television	168 (55.6)	116 (76.8)	102 (55.1)	76 (83.5)
	Mobile phone with internet	53 (17.6)	61 (40.4)	57 (30.8)	32 (35.2)
	Mobile phone without internet	268 (88.7)	121 (80.1)	173 (93.5)	86 (94.5)
**Birth outcome at posttest, n (%)**					
	Born alive and healthy	272 (89.2)	138 (95.6)	165 (89.2)	87 (95.6)
	Stillbirth	13 (4.9)	4 (1.1)	9 (4.9)	1 (1.1)
	Other^c^	17 (6.0)	9 (3.3)	11 (6.0)	3 (3.3)

^a^MAMA: Mobile Alliance for Maternal Action.

^b^Multiple responses allowed.

^c^Other responses included abortion, miscarriage, and women who had not yet given birth.

### MAMA Program Feasibility

The majority (312/453, 68.9%) of female participants overall reported that they had to seek the permission of a gatekeeper to enroll in the MAMA program; this gatekeeper was usually the woman’s husband, although in some cases it was the mother-in-law (Table 2). Some subscribers reported not being offered a preferred time to receive messages by the community health worker during MAMA registration, but most of the women who chose a time preferred to receive messages in the morning or at night (Table 2).

After 6 months of program participation, the automated database confirmed that across groups, 95%-97% of respondents had not cancelled their subscription and were continuing to receive messages (Table 2). Of the 22 women and 8 men who were no longer subscribers at follow up, the majority (21/30, 70%) did not have the phone or SIM card used at registration or it was broken; a few women (3/30, 10%) had problems related to phone sharing. The program was designed to send 2 messages each week, with a few exceptions; however, some respondents reported receiving more than 2 messages per week. A software error that was not detected during beta testing caused the automated system to erroneously send multiple messages per day to the same phone number. Affected subscribers notified the project through a helpline or by telling the community health workers who then called HEMAYAT project staff to report the error. This problem was identified and corrected within 48 hours of reporting. Subscribers of voice messages received fewer total messages and were more likely to report occasionally missing messages compared to those who received text messages; women also stated more often than men that they missed messages. Common reasons for missing messages included being busy with chores or that someone else had the phone, and these reasons were equally common for both sexes.

### MAMA Program Acceptability

Most women and men cited multiple benefits when asked about perceived program benefits (Table 3); gaining information about health care for themselves (women), their wives (men), or their children were the most frequently stated benefits. Women often reported that the ability to obtain health information at home was also a benefit, and about one-quarter of the respondents of both sexes spontaneously mentioned that they learned new health information by subscribing to MAMA. Nearly all men and over 80% of women (n=377) agreed that it was beneficial to include husbands in the MAMA program; the majority of women in the voice message group and approximately half of the women in the SMS text messaging group said their husbands had listened to or read the messages (Table 3). More than half of the participants agreed that including their mothers-in-law was beneficial, but 119 women (about 25%) stated that it was not beneficial. Most women reported having discussed the program with a peer, with about half of women and over one-third of men reporting having recommended the program to a friend or relative.

**Table 2:** Feasibility of Mobile Alliance for Maternal Action (MAMA) program registration and use among participants, by sex and message modality, in four provinces of Afghanistan (N=729).

Feasibility measure		Women		Men	
		Voice (n=302)	Text messaging (n=151)	Voice (n=185)	Text messaging (n=91)
Woman had to ask permission to register^a^		213 (70.5)	99 (65.6)	131 (70.8)	47 (51.7)
**Who was asked for permission^b^**					
	Husband	174 (78.4)	83 (79.8)	96 (69.6)	34 (66.7)
	Mother-in-law	32 (14.4)	18 (17.3)	34 (24.6)	14 (27.5)
	Father-in-law	13 (5.9)	3 (2.9)	8 (5.8)	3 (5.9)
	Brother-in-law	2 (0.9)	0 (0)	0 (0)	0 (0)
	Other (specify):	1 (0.45)	0 (0)	0 (0)	0 (0)
**Received message at preferred time at baseline, n (%)**					
	Not offered the choice of a preferred time	101 (33.4)	39 (25.8)	40 (21.6)	18 (19.8)
	Yes	136 (45.0)	89 (58.9)	76 (41.1)	38 (41.8)
	No	49 (16.2)	18 (11.9)	11 (5.9)	9 (9.9)
	Refused or don’t know	16 (5.3)	10 (6.6)	58 (31.4)	26 (28.6)
**Time of day preferred (baseline), n (%)**					
	Morning	88 (29.1)	43 (28.5)	40 (21.6)	23 (25.3)
	Afternoon	27 (8.9)	18 (11.9)	18 (9.7)	8 (8.8)
	Evening	31 (10.3)	9 (6)	17 (9.2)	2 (2.2)
	Night	55 (18.2)	39 (25.8)	12 (6.5)	7 (7.7)
	Anytime	0 (0)	3 (2)	9 (4.9)	9 (9.9)
	No choice given/don’t know	101 (33.4)	39 (25.8)	89 (48.1)	42 (46.2)
Current MAMA subscription at posttest^c^, n (%)		286 (94.7)	145 (96)	180 (97.3)	88 (96.7)
Total messages received, mean (SD)		43 (17.6)	69.4 (13)	43.7 (17.7)	68.5 (12.5)
**Typical number messages received per week^d^, n (%)**					
	0	2 (0.7)	2 (1.3)	5 (2.7)	1 (1.1)
	1-2	240 (79.5)	137 (90.7)	104 (56.2)	62 (68.1)
	3-5	47 (15.6)	12 (8)	63 (34.1)	25 (27.5)
	6+	13 (4.3)	0 (0)	13 (7)	3 (3.3)
Ever missed messages, n (%)		142 (47)	36 (23.8)	49 (26.5)	14 (15.4)
**Reasons for missing messages, among those who reported missing^b^, n (%)**		N=142	N=36	N=49	N=14
	No balance or charge	14 (7.7)	1 (2.3)	13 (17.8)	4 (19.1)
	Busy with chores	73 (40.3)	18 (41.9)	37 (50.7)	8 (38.1)
	Someone else had phone	67 (37)	18 (41.9)	19 (26)	7 (33.3)
	Wrong time	7 (3.9)	0 (0)	0 (0)	1 (4.8)
	Someone else took the call	5 (2.8)	3 (7)	0 (0)	0 (0)
	Other	15 (8.3)	3 (7)	4 (5.5)	1 (4.8)

^a^Male participants were asked whether their wives were required to obtain permission to register.

^b^Multiple responses allowed.

^c^Subscription and messages received data were extracted from the MAMA system database. All other data presented were based on self-reporting.

^d^Men were asked how many messages their wives received per week.

**Table 3:** Mobile Alliance for Maternal Action (MAMA) program acceptability among participants, by sex and message type, in four provinces of Afghanistan (N=729).

Acceptability measure			Women			Men			
		Voice (n=302)		Text messaging (n=151)		Voice (n=185)		Text messaging (n=91)	
**Reported benefits of MAMA^a^, n (%)**									
	Information for own (wife’s) health	218 (72.2)		108 (71.5)		109 (58.9)		60 (65.9)	
	Information for child’s health	201 (66.6)		107 (70.9)		114 (61.6)		58 (63.7)	
	Able to get health information at home	121 (40.1)		47 (31.1)		56 (30.3)		23 (25.3)	
	Learned new health information	85 (28.2)		39 (25.8)		47 (25.4)		14 (15.4)	
	Other	11 (3.6)		2 (1.3)		10 (5.4)		8 (8.8)	
	None	10 (3.3)		4 (2.7)		8 (6)		0 (0)	
**Others in household have listened to/read messages, n (%)**									
	No one	79 (26.2)		53 (35.1)		116 (62.7)		64 (70.3)	
	Husband	182 (60.3)		73 (48.3)		N/A^b^		N/A	
	Mother-in-law/Mother (for husband)	76 (25.2)		16 (10.6)		47 (25.4)		17 (18.7)	
	Sister(s)-in-law	28 (9.3)		14 (9.3)		9 (4.9)		6 (6.6)	
	Sister(s)	26 (8.6)		9 (6)		12 (6.5)		14 (15.4)	
	Father-in-law/father (for husband)	19 (6.3)		2 (1.3)		14 (7.6)		3 (3.3)	
	Other	12 (4)		2 (1.3)		17 (9.2)		5 (5.5)	
**Agree that including husband is beneficial, n (%)**									
	Yes	256 (84.8)		121 (80.1)		173 (93.5)		85 (93.1)	
	No	31 (10.3)		21 (13.9)		4 (2.2)		2 (2.2)	
	Don’t know	15 (5)		9 (6)		8 (4.3)		4 (4.4)	
**Agree that including mother-in-law (husband’s mother) in program is beneficial, n (%)**									
	Yes	151 (50)		77 (51)		114 (61.6)		62 (68.1)	
	No	76 (25.2)		43 (28.5)		38 (20.5)		15 (16.5)	
	Don’t know	29 (9.6)		6 (4)		33 (17.8)		14 (15.4)	
	Mother-in-law didn’t listen to messages	46 (15.2)		25 (16.7)		N/A		N/A	
**Benefits of including mother-in-law^a^, n (%)**									
	Mother-in-law helps participant understand messages	89 (29.5)		52 (34.4)		65 (35.1)		36 (39.6)	
	Helps participant follow instructions in messages	48 (15.9)		25 (16.6)		35 (18.9)		27 (29.7)	
	Increased mother-in-law’s awareness of participant’s health needs	64 (21.2)		26 (17.2)		63 (34.1)		21 (23.1)	
	Needs information on health care during pregnancy	68 (22.5)		17 (11.3)		25 (13.5)		8 (8.8)	
	Other	1 (0.3)		2 (1.3)		1 (0.5)		3 (3.3)	
Discussed MAMA with other pregnant women or new mothers, n (%)		175 (58)		88 (58.3)		N/A		N/A	
Recommended the MAMA program, n (%)		145 (48)		81 (53.6)		67 (36.2)		39 (42.9)	
**Who MAMA was recommended to^a^, n (%)**									
	Family member	66 (45.5)		41 (50.6)		24 (35.8)		17 (43.6)	
	Friend	55 (37.9)		32 (39.5)		42 (62.7)		17 (43.6)	
	Neighbor	68 (46.9)		31 (38.3)		39 (58.2)		12 (30.8)	
	Others	3 (2.1)		0		2 (3.0)		3 (7.7)	

^a^Multiple responses possible.

^b^N/A: not applicable; option/item not asked of men.

### Attitudes, Decision Making, and Knowledge

We assessed attitudes toward MNCH, including comfort discussing maternal and newborn care with family members, correct knowledge and acceptance of exclusive breastfeeding and birth spacing, and attitudes about utilizing facility-based care during pregnancy and delivery (Table 4). The proportion of participants who agreed that they feel knowledgeable discussing health care for pregnancy and newborns with their spouse was high at baseline and increased slightly at follow up; however, there was little or no change in women feeling knowledgeable in discussing pregnancy or infant care with their mothers-in-law. Participant attitudes about breastfeeding and birth spacing improved between measures for both women and men; however, 118 (42.8%) of men at follow up still agreed that exclusive breastfeeding will not result in appropriate infant growth. The proportion of respondents who agreed that their mother-in-law or mother (for men) supported delivering their babies in a health facility decreased at posttest.

With respect to decision making about MNCH care, and when to seek services, reported joint decision making by respondents and their spouses together increased between time points (Table 5). In general, there was a wide range of responses to questions about who makes health care decisions with “respondent,” “spouse,” “respondent and spouse jointly,” and “mother-in-law/mother” all being common choices.

We examined differences between baseline and follow up in participants’ MNCH knowledge that overlapped with the MAMA message content. Generally, knowledge was higher for all indicators among women compared to men at both time points (Table 6). Knowledge for all measured topics increased between time points for both sexes with one exception, knowledge of the maximum time the lactational amenorrhea method has a reliable contraceptive effect; knowledge differences between women and men diminished on several items at follow up. Knowledge of applying chlorhexidine to a newborn’s umbilical cord stump improved substantially and knowing to start breastfeeding within 1 hour after delivery improved substantially among men.

**Table 4:** Attitudes toward maternal, newborn, and child health care at baseline and follow up, by participant sex, in four provinces of Afghanistan (N=729).

Item		Women (n=453)		Men (n=276)	
		Baseline, n (%)	Follow up, n (%)	Baseline, n (%)	Follow up, n (%)
**Feel knowledgeable discussing health care for pregnancy with spouse**					
	Agree	395 (87.2)	405 (89.4)	228 (82.6)	247 (89.5)
	Neutral	33 (7.3)	25 (5.5)	21 (7.6)	18 (6.5)
	Disagree	19 (4.2)	20 (4.4)	10 (3.6)	5 (1.8)
	Refused	6 (1.3)	3 (0.7)	17 (6.2)	6 (2.2)
**Feel knowledgeable discussing health care for baby with spouse**					
	Agree	395 (87.2)	413 (91.2)	224 (81.2)	248 (89.9)
	Neutral	33 (7.3)	24 (5.3)	28 (10.1)	13 (4.7)
	Disagree	19 (4.2)	14 (3.1)	12 (4.4)	11 (4)
	Refused	6 (1.3)	2 (0.4)	12 (4.4)	4 (1.5)
**Feel knowledgeable discussing pregnancy health care with mother-in-law (mother)**					
	Agree	333 (73.5)	334 (73.7)	206 (74.6)	222 (80.4)
	Neutral	42 (9.3)	62 (13.7)	29 (10.5)	11 (4)
	Disagree	35 (7.7)	32 (7.1)	21 (7.6)	29 (10.5)
	Refused	43 (9.5)	25 (5.5)	20 (7.3)	14 (5.1)
**Feel knowledgeable discussing baby health care with mother-in-law (mother)**					
	Agree	333 (73.5)	327 (72.2)	215 (77.9)	219 (79.4)
	Neutral	42 (9.3)	56 (12.4)	27 (9.8)	11 (4)
	Disagree	36 (8)	43 (9.5)	18 (6.5)	33 (12)
	Refused	42 (9.3)	27 (6)	16 (5.8)	13 (4.7)
**Believe it is against Islam to use birth-spacing methods**					
	Agree	94 (20.8)	67 (14.8)	73 (26.5)	35 (12.7)
	Neutral	31 (6.8)	12 (2.7)	26 (9.4)	17 (6.2)
	Disagree	310 (68.4)	359 (79.3)	158 (57.3)	205 (74.3)
	Refused	18 (4)	15 (3.3)	19 (6.9)	19 (6.9)
**Believe exclusive breastfeeding is inadequate; babies need supplemental foods/liquids**					
	Agree	171 (37.8)	76 (16.8)	138 (50)	118 (42.8)
	Neutral	44 (9.7)	31 (6.8)	29 (10.5)	39 (14.1)
	Disagree	220 (48.6)	341 (75.3)	95 (34.4)	113 (40.9)
	Refused	18 (4)	5 (1.1)	14 (5.1)	6 (2.2)
**Believe mother-in-law (mother) supports health facility delivery**					
	Agree	360 (79.5)	340 (75.1)	236 (85.5)	226 (81.9)
	Neutral	26 (5.7)	47 (10.4)	11 (4)	13 (4.7)
	Disagree	32 (7.1)	40 (8.8)	19 (6.9)	16 (5.8)
	Refused	35 (7.7)	26 (5.7)	10 (3.6)	21 (7.6)

**Table 5:** Reported health decision makers within the household among participants, by sex, across four provinces in Afghanistan (N=729).

Health decision maker		Women (n=453)		Men (n=276)	
		Baseline, n (%)	Follow up, n (%)	Baseline, n (%)	Follow up, n (%)
**Regarding mother’s health**					
	Respondent	100 (22.1)	83 (18.3)	65 (23.6)	79 (28.6)
	Spouse	151 (33.3)	139 (30.7)	41 (14.9)	29 (10.5)
	Respondent and spouse jointly	73 (16.1)	114 (25.2)	89 (32.3)	104 (37.7)
	Mother-in-law/mother	80 (17.7)	76 (16.8)	57 (20.7)	42 (15.2)
	Husband/wife and mother-in-law/mother jointly	18 (4)	8 (1.8)	4 (1.5)	0 (0)
	Other relative	27 (6)	33 (7.3)	17 (6.2)	21 (7.6)
	No response/refused	4 (0.9)	0 (0)	3 (1.1)	1 (0.4)
**Regarding infant/child health**					
	Respondent	106 (23.4)	110 (24.3)	54 (19.6)	49 (17.8)
	Spouse	125 (27.6)	109 (24.1)	60 (21.7)	49 (17.8)
	Respondent and spouse jointly	93 (20.5)	127 (28)	89 (32.3)	116 (42)
	Mother-in-law/mother	86 (19)	73 (16.1)	55 (19.9)	41 (14.9)
	Husband/wife and mother-in-law/mother jointly	21 (4.6)	3 (0.7)	2 (0.7)	4 (1.5)
	Other relative	16 (3.5)	31 (6.8)	15 (5.4)	17 (6.2)
	No response/refused	6 (1.3)	0 (0)	1 (0.4)	0 (0)
**Regarding care-seeking**					
	Respondent	82 (18.1)	81 (17.9)	90 (32.6)	74 (26.8)
	Spouse	214 (47.2)	152 (33.6)	28 (10.1)	27 (9.8)
	Respondent and spouse jointly	64 (14.1)	115 (25.4)	93 (33.7)	118 (42.8)
	Mother-in-law/mother	48 (10.6)	64 (14.1)	34 (12.3)	28 (10.1)
	Husband/wife and mother-in-law/mother jointly	11 (2.4)	12 (2.7)	2 (0.7)	4 (1.5)
	Other relative	31 (6.8)	29 (6.4)	27 (9.8)	24 (8.7)
	No response/refused	3 (0.7)	0 (0)	2 (0.7)	1 (0.4)

**Table 6:** Comparison of reported maternal, newborn, and child health care awareness and knowledge differences by time point and sex across four provinces in Afghanistan with the paired McNemar Chi square test (N=729).

Item	Women (n=453)			Men (n=276)			
	Baseline, n (%)	Follow up, n (%)	*P* value		Baseline, n (%)	Follow up, n (%)	*P* value
Knew any reason to take iron supplements	265 (58.5)	377 (83.2)	<.001		129 (46.7)	161 (58.3)	.004
Knew ≥4 antenatal care visits recommended	297 (65.6)	345 (76.2)	<.001		164 (59.4)	188 (68.1)	.03
Knew ≥1 pregnancy warning signs	382 (84.3)	436 (96.3)	<.001		197 (71.4)	246 (89.1)	<.001
Knew ≥1 childbirth warning signs	395 (87.2)	433 (95.6)	<.001		211 (76.5)	252 (91.3)	<.001
Knew ≥1 way to keep baby warm	351 (77.5)	427 (94.3)	<.001		185 (67.0)	242 (87.7)	<.001
Knew to apply chlorhexidine to cord	201 (44.4)	303 (66.9)	<.001		80 (29.0)	183 (66.3)	<.001
Knew when to start breastfeeding	400 (88.3)	422 (93.2)	.01		151 (54.7)	222 (80.4)	<.001
Knew recommended duration to practice exclusive breastfeeding	392 (86.5)	427 (94.3)	<.001		130 (47.1)	207 (75.0)	<.001
Knew maximum time LAM^a^ can be practiced	63 (13.9)	57 (12.6)	.50		22 (8.0)	39 (14.1)	.02

^a^LAM: lactational amenorrhea method.

## Discussion

### Principal Results

Our results indicate that the adapted MAMA program was feasible and acceptable to implement in Afghanistan. Consistent with other evidence from Afghanistan, most participants preferred to receive voice calls rather than SMS text messages [16]. Nearly all participants continued to receive messages 6 to 8 months after enrollment and reported that they benefitted from the program. Women who received voice messages more often reported that their husband or mother-in-law engaged with the messages compared to women who received text messages. Text message recipients received more messages on average than those who received voice messages, but this is explained by the fact that the character limit for text messages required some MAMA messages to be divided into two or three separate texts. In open-ended responses, participants who said that they had missed messages or were no longer subscribers usually cited reasons related to not having the phone or SIM card that was used to enroll in the program. Phone sharing within households also appears to have contributed to missed messages. However, at follow up, less than 3% of women and men stated they were not receiving any messages on a weekly basis, suggesting technical viability of the program in this and possibly other fragile contexts. Men reported that they felt more knowledgeable discussing MNCH topics at follow up. Reported joint decision making between spouses about MNCH care modestly increased. Knowledge of several lifesaving MNCH issues and interventions, including pregnancy warning signs, newborn umbilical cord care, and breastfeeding, improved over the 6-month assessment period among both women and men.

### Comparison With Prior Work

The proliferation of mobile phones is an important development in the Afghan context [4,28]; however, phone sharing practices, low literacy, a weak communications infrastructure, and social norms that limit women’s ability to communicate, make, and act on health decisions are all factors that may hinder the success of an mHealth program in such a context. This study is one of few conducted in fragile settings such as Afghanistan to examine the feasibility and acceptability of using mobile phones for health education and to promote behavior change. Moreover, despite evidence of the primacy of male heads-of-household in health decision making [13], this is the only study conducted in Afghanistan to date that describes the implementation of an mHealth program that includes both women and men.

A recent review found mixed results and insufficient evidence that mHealth interventions improve MNCH outcomes [29]. However, many studies have tried to assess changes in health outcomes such as rates of antenatal care attendance, as opposed to more proximal measures such as knowledge and attitudes [26]. Additionally, most interventions have focused on only one stage of MNCH care (pregnancy, birth, postpartum, or infant care) [29], whereas the present evaluation spans the pregnancy and postpartum periods.

Another unique aspect of this study is the comparison of voice to SMS text message formats regarding participant characteristics, feasibility, and acceptability measures. In several studies conducted in a variety of contexts, voice calls were preferred over SMS text messages [30]; however, only one study conducted in Malawi compared SMS text and voice messages for improving the knowledge and uptake of maternal and newborn health practices [21]. The authors found that SMS text messages had a higher rate of successful message delivery and SMS participants were significantly more likely to report a behavior change compared to voice message participants; however, some participants could only access voice messages due to low literacy. In our study, a surprisingly large number of MAMA subscribers opted to receive text messages, despite low literacy. Additional research is needed to better understand the factors influencing message format selection and the benefits and limitations of each of these methods of message delivery in this context.

Two papers have presented findings from different analyses of the MAMA program in Bangladesh, called Aponjon, which is operated by the nonprofit social enterprise Dnet [24,25]. The first reported results were from a cross-sectional survey conducted among 255 women who had subscribed to Aponjon during pregnancy for at least 3 months and 389 matched controls for each subscriber, as well as 345 women who subscribed to Aponjon during the postpartum period and 455 matched nonsubscribers [24]. The researchers compared results on >75 knowledge and behavioral indicators between exposed and nonexposed women, finding statistically significant differences for 24 of the indicators. However, there was no clear pattern in the results to indicate an association between a specific knowledge area or behavior and the intervention. The authors reported that when comparing degrees of exposure (categorized as nonexposure, 3-5 months exposure, and 6-12 months exposure), they observed statistically significant associations between 6-12 month exposure and scoring in the 50th percentile or greater on composite measures of newborn knowledge and newborn health practices compared to those with no program exposure [25]. The second paper reported the results of a subanalysis comparing perinatal outcomes between women who started using the Aponjon program during pregnancy with those of women who enrolled in Aponjon postpartum. The authors found that exposure to program messages during pregnancy was not associated with skilled birth attendance, initiating breastfeeding within 2 hours after delivery, timing of the first bath for the baby, or frequency of postnatal care visits [24]. Although our study design precludes attributing changes in knowledge or attitudes to the intervention, the changes observed among both sexes are promising and warrant further study with research designed to test the effectiveness of the intervention on knowledge and behavioral outcomes.

### Limitations

There are several limitations of this study. We acknowledge that the single-group study design precludes attributing changes in knowledge, attitudes, and decision making to the MAMA intervention, and therefore we treat these results as exploratory. The ability to generalize our findings is also limited. Those who opted to subscribe to the MAMA program may differ substantially from those who opted not to subscribe; however, community health workers reported that very few of the women they approached did not subscribe. Additionally, our analyses included only 55% of women who subscribed to the pilot program; therefore, these results may not reflect subscribers as a whole. Participants who could be located and consented to be interviewed at both time points may have had higher engagement and more positive views of the program. Our measurement of MNCH knowledge items reflects participant knowledge at different stages of partial exposure to the program. Because the baseline interview was administered in some cases up to 10 weeks after enrollment and participants were at various stages of pregnancy, some participants were already exposed to some of the knowledge items we assessed at baseline, while others were not. At follow up, participants had similarly varying exposure to newborn care messages. Similarly, due to phone sharing within households, we elected not to assess whether knowledge change was proportionate to the number of messages received. Finally, there may have been confounding effects through exposure to other MNCH information channels during the pilot program. Community health workers at the community level and health care providers are the primary sources of MNCH messages and education in rural Afghanistan, although some households may also receive health information from other community-level volunteers (eg, Family Health Action Group members), radio, or television [31,32]. We are unaware of any other new health education programs implemented in the same communities at the same time as the MAMA pilot program. The existing sources may have created some bias, which we cannot rule out due to lack of a comparator group.

### Conclusions

We found that Afghan women and men welcomed an mHealth MNCH educational program and were largely able to enroll and access weekly voice or text messages with few technological complications, and continued program use from pregnancy through early infancy. Participants reported that they benefited by gaining health knowledge, and many stated that they discussed the messages with their family and peers. Joint decision making between spouses appears to have increased during the intervention period, and including husbands in the program was more often described as beneficial by participants when compared with the discussion of messages with mothers-in-law; both are potential areas for further research in this cultural context. There were improvements in MNCH knowledge measures among both male and female participants; however, given the important limitations of the research that has been conducted on this approach, future research of this and similar programs should be designed to rigorously evaluate the effect of the intervention on MNCH knowledge and health behaviors. Prior to further effectiveness evaluation, we hope that the data from this assessment will be used to secure ongoing and sustained support to allow the MAMA program to be refined and implemented at a greater scale in Afghanistan.

## Abbreviations

HEMAYAT: Helping Mothers and Children Thrive in Afghanistan
MAMA: Mobile Alliance for Maternal Action
mHealth: mobile health
MNCH: maternal, newborn, and child health
MOPH: Ministry of Public Health
SMS: short message service
USAID: United States Agency for International Development
